# Dr. Marsden’s Plan of Quarantine

**Published:** 1866-02

**Authors:** 


### Dr. Marsden's Plan of Quarantine.

It may suffice, perhaps, to state that the plan of quarantine proposed by Dr. Marsden contemplates three separate and distinct sections or departments; each being isolated and separated from one another by a *cordon*, or portion of neutral ground, of not less than one hundred feet wide. One of three sections or departments to be appropriated to the use of the sick, and called the *hospital department*. The next, or central section, is to be devoted to the use of passengers not having had cholera, but from infected vessels. The third, or healthy department, to be appropriated to the use of the healthy, who had been removed from the central department, ofter having performed quarantine there.

In the *first section or department*, there are to be three distinct and separate hospitals, besides a convenient shed or hospital; the one for confirmed cases of cholera, to be called the CHOLERA HOSPITAL. Another for cases of choleraic diarrhœa, or other premonitory symptoms of cholera, to be called the HOSPITAL FOR CHOLERINE. The third, for all other diseases, not cholera, or cholerine, but coming from on board infected vessels, or vessels having had cases of cholera on board, to be called the GENERAL HOSPITAL.

The central section or department, is to be the primary quarantine department, and appropriated to all persons who are not sick, but came from vessels having had cholera on board, and wherein every case on landing shall undergo inspection, washing, cleansing and purifying, both of persons and personal effects. There a quarantine of four days is to be performed, at the end of which period of time all such persons as continue in sound health, shall be removed to the FINAL QUARANTINE DEPARTMENT, and any that may fall sick or threatened with sickness during the four days of probation, shall, as soon as detected, be removed to the proper hospital, in the Hospital Department. There, also, the healthy inmates shall be removed daily to a new locality, thus occupying four different habitations during their sojourn.

The third, or healthy department, is to be the final department, and intended for all cases coming from the primary quarantine department, after having been cleansed, washed and disinfected, and after having undergone the *four days* quarantine; and here a further quarantine of *six days* is to be performed (excepting cases coming from the convalescent hospital or shed, hereinafter provided for) making in all *ten days* of quarantine, when all persons continuing healthy, shall be discharged from quarantine, and be removed from the station. If any premonitory symptoms or other cases of sickness occur in this department during the six days of quarantine, they are, as soon as discovered, to be removed to the proper hospital, in the Hospital Department.

No communication shall take place with the Hospital Department, except through the central or Primary Quarantine Department, for which purpose a passage, unfrequented by the persons undergoing quarantine, shall be set apart and reserved.

The three sections or departments above described shall be designated and known as—

1.—The Hospital Department.

2.—The Primary Quarantine Department.

3.—The Final Quarantine Department.

To Pilots.—1. All vessels coming from infected ports, and having, or having had cholera cases on board, shall be brought to anchor abreast of the central or Primary Quarantine Department or Station.

2. All vessels coming from ports known to be infected by cholera, or not, and not having or having had any case or cases of cholera on board, shall be brought to anchor abreast of the healthy or Final Quarantine Department or Station, where, and when, they shall be boarded by the Medical Officer of that Department, and he shall have power either to discharge them from quarantine forthwith, or detain them, if he finds sufficient cause for so doing.

OF LANDING AND RE-EMBARKING.

*a.* The landing of passengers and their effects shall take place at the Primary Quarantine Department *only*.

*b.* The re-embarking of passengers and their effects shall take place from the Final Quarantine Department *only*.

1.—On the landing of passengers from on board ship at the Primary Quarantine Station, the sick shall be forthwith removed to the Hospital Department, and the healthy to the place assigned to them, in the Primary Quarantine Department.

2.—The sick shall be borne upon litters and placed within the neutral limits about midway between the Primary Quarantine and Hospital Departments, by the persons who bring them ashore, and who shall then retire to the Primary Quarantine Department, (unless they be seamen belonging to the vessel, in which case they shall return aboard ship;) whereupon persons from the Hospital Department shall enter the neutral ground, and remove them to the proper hospital.

3.—There shall be in the Hospital Department, at a reasonable distance from the Cholera Hospital, a shed or building for cholera convalescents, where they shall remain at least for *four days* previous to being removed to the Primary Quarantine Department, and where a quarantine of *four more days* shall be performed after cleansing, washing and purifying, previous to removal to the Final Quarantine Department, where *two more days* of quarantine only, instead of six, shall be performed, making in all *ten clear days* after leaving the Cholera Hospital, when, if the patient continues healthy, he or she shall be discharged.

4.—Persons having completed their period of quarantine shall be removed at once from the Quarantine Station, by steamers chartered for the purpose, and shall proceed directly on their journey.

5.—Provisions, stores, clothing, bedding, and all other necessaries or supplies for the Hospital Department shall be conveyed within the hospital limits, under the same regulations and restrictions as persons.

6.—All physicians, orderlies, servants, nurses, attendants, etc., connected with the Cholera Quarantine Station, as also all persons performing quarantine, shall remain and be kept constantly in the department or section to which they have respectively been assigned, and none of them shall, under any pretext whatever, be permitted to have any communication or intercourse whatever, directly or indirectly, with persons from another department or section, excepting in due course of quarantine.

7.—Any employee, nurse or orderly belonging to the Quarantine Station, who may be found violating the above rule, shall be liable to suspension from office, with forfeiture of salary and emoluments, or dismissal from office, at the discretion of the Medical Officer in charge, or of the Superintendent, besides being obliged to undergo such quarantine as the nature of the contact or exposure may warrant.

8.—Any person violating the above rule by going from the Final Quarantinc Department to the Primary Quarantine Department, or from either of these to the Hospital Department, shall, on detection, be detained in the department they have gone into, in violation of the law, and shall undergo quarantine there anew.

9.—All persons suffering the approach of persons from another department, except in due course of quarantine, will render themselves liable at the discretion of the medical officer, to be sent back to the department to which the person so approaching them belonged, and shall undergo quarantine anew.

10.—The three Quarantine Sections or Departments shall be separated from each other, and bounded by a *cordon* or piece of neutral ground, of at least one hundred feet in width, and shall be surrounded by a strong fence of at least seven feet high.

11.—Between the Final Quarantine and Hospital Departments, at the extreme end of the Primary Quarantine Department, there shall be a cordon, or passage or portion of ground, of at least thirty feet wide, with a close fence of seven feet high, to be used exclusively as a passage from the Final to the Hospital Department, for the return of patients to the Hospital Department if necessary.

12.—Each of the sub-divisions in the Hospital Department shall be surrounded by an open fence of seven feet high.

13.—Each of the sub-divisions in the other departments, and especially in the Primary Quarantine Department, shall be surrounded by a close fence of seven feet high.

14.—Each of the before mentioned departments may and shall be sub-divided, in such manner as circumstances may require; and as near as practicable in accordance with the accompanying plan.

15.—The place of landing in the Primary Quarantine Department shall be as near the Hospital Department as convenient, and as far removed as possible from the place of departure or re-embarkation, in the Final Quarantine Department.

16.—There shall be telegraphic communication between each of the departments, with a telegraph operator attached to each.

Among the additional details of my plan, the following is most important:

A *perpetual* stream of water shall be made to flow through all the water-closets, cess-pools, drains, etc., which shall empty themselves at low water-mark; and such other disinfectants and deodorizers as science may suggest and necessity dictate, shall also be used.

W. MARSDEN, M. D.

We perceive from a recent New York paper that the above plan of quarantine of Dr. Marsden, was recently presented to the Board of Health of that city, with a recommendation that the Board request the General Government to adopt said plan, with some slight improvements, and make its enforcements compulsory at every port of entry on the entire coast.

